# Putative psychosis genes in the prefrontal cortex: combined analysis of gene expression microarrays

**DOI:** 10.1186/1471-244X-8-87

**Published:** 2008-11-07

**Authors:** Kwang Ho Choi, Michael Elashoff, Brandon W Higgs, Jonathan Song, Sanghyeon Kim, Sarven Sabunciyan, Suad Diglisic, Robert H Yolken, Michael B Knable, E Fuller Torrey, Maree J Webster

**Affiliations:** 1 Stanley Laboratory of Brain Research, 9800 Medical Center Dr. Bldg 2C, Rockville, MD 20850, USA; 2 Elashoff Consulting, Redwood City, CA 94065, USA; 3 Stanley Laboratory of Developmental Neurovirology, Johns Hopkins University, School of Medicine, 600 North Wolfe Street, Blalock 1105, Baltimore, MD 21287, USA; 4 Stanley Medical Research Institute, 8401 Connecticut Ave, Suite 200, Chevy Chase, MD 20815, USA

## Abstract

**Background:**

Recent studies have shown similarities between schizophrenia and bipolar disorder in phenotypes and in genotypes, and those studies have contributed to an ongoing re-evaluation of the traditional dichotomy between schizophrenia and bipolar disorder. Bipolar disorder with psychotic features may be closely related to schizophrenia and therefore, psychosis may be an alternative phenotype compared to the traditional diagnosis categories.

**Methods:**

We performed a cross-study analysis of 7 gene expression microarrays that include both psychosis and non-psychosis subjects. These studies include over 400 microarray samples (163 individual subjects) on 3 different Affymetrix microarray platforms.

**Results:**

We found that 110 transcripts are differentially regulated (p < 0.001) in psychosis after adjusting for confounding variables with a multiple regression model. Using a quantitative PCR, we validated a set of genes such as up-regulated metallothioneins (MT1E, MT1F, MT1H, MT1K, MT1X, MT2A and MT3) and down-regulated neuropeptides (SST, TAC1 and NPY) in the dorsolateral prefrontal cortex of psychosis patients.

**Conclusion:**

This study demonstrates the advantages of cross-study analysis in detecting consensus changes in gene expression across multiple microarray studies. Differential gene expression between individuals with and without psychosis suggests that psychosis may be a useful phenotypic variable to complement the traditional diagnosis categories.

## Background

Kraepelin's 1896 dichotomous classification of functional psychoses into dementia praecox (schizophrenia) and manic-depressive insanity (bipolar disorder) is well known and has dominated most diagnostic systems in psychiatry. Less well known is the fact that two decades later, Kraepelin himself questioned the validity of his classificatory system, noting that "it is becoming increasingly clear that we cannot distinguish satisfactorily between these two illnesses and this brings home the suspicion that our formulation of the problem may be incorrect." [1,2] In recent years, increasing number of observers have shared Kraepelin's doubts, with some proposing instead a continuum hypothesis [3,4]. Clinically, there is much overlap of symptoms, and the intermediate category of schizoaffective disorder may imply an artificial preservation of the dichotomy between these two diagnoses [5]. The clinical gulf between individuals with bipolar II and those with bipolar I with psychotic features is marked, whereas the latter often resemble individuals with schizophrenia quite closely. Epidemiologically, there is also some overlap, especially in so far as both have a winter-spring seasonal birth excess [6,7].

Genetically, it has been said that "accumulating evidence supports the existence of an overlap in genetic susceptibility across the traditional Kraepelinian divide" [8]; this has been reported for family studies, linkage studies, and candidate genes such as G72, NRG1, dysbindin, COMT, BDNF, and DISC1 [9-12]. An overlap for some neuropsychological dysfunctions, such as working memory [13], has also been reported, although individuals with bipolar disorder are less severely impaired [14]. This suggests that working memory dysfunction may be closely related to psychotic features in individuals with schizophrenia and with bipolar disorder.

Neuropathologically, an overlap in findings between schizophrenia and bipolar disorder has also been noted, both neurochemically [15] and structurally [16]. As was true for the neuropsychology findings, individuals with bipolar disorder are structurally less severely impaired. Neuropathological studies that have divided bipolar patients into those with and without psychotic features have reported that the former are more closely related to schizophrenia than the latter [17]. This last observation suggests that an important variable in categorizing seriously mentally ill individuals may be the presence or absence of psychotic features. To date, most studies of gene expression in schizophrenia and bipolar disorder have used the traditional Kraepelinian classification. We, therefore, decided to reanalyze a set of microarray studies, dividing the individuals within each study into those with and without psychotic features.

Although genome-wide expression studies of psychiatric disorders have been published, there have been inconsistent findings among previous studies possibly due to several factors including: relatively small sample sizes, inappropriate p-value and fold change criteria, and failure to adjust for potential confounding variables. In order to address these issues, we performed a cross-study analysis of 7 microarray datasets using the subjects with and without psychotic features from the two post-mortem brain collections of the Stanley Medical Research Institute (SMRI). The microarray datasets of the prefrontal cortex with three Affymetrix platforms (hgu133A, hgu133 2.0+ and hgu95Av2) were used for this cross-study analysis.

## Methods

### Post-mortem brain tissues

Two post-mortem brain collections from the SMRI have been made available to researchers worldwide. The first, the Neuropathology Consortium, has 60 individual subjects, with multiple brain regions per subject. The details of the sample collection have been described previously [18]. Notable exclusion criteria include: age>65 years, poor quality mRNA, and significant structural brain pathology on post-mortem examination. These samples are matched for age, gender, race, pH, post-mortem interval (PMI), side of the brain and mRNA quality. For microarray studies using this collection, tissue samples from the prefrontal cortex (Brodmann area 46/10, 6 and 8/9) were provided to individual investigators, who then performed the RNA extraction and experiment.

The second, the Array Collection, consists of 105 individual subjects, with multiple brain regions per subject. Exclusion criteria are similar to those for the Neuropathology Consortium. For this collection, dorsolateral prefrontal cortex (Brodmann area 46) was used for the microarrays. In contrast to the Neuropathology Consortium, the SMRI performed the RNA extraction for the Array Collection tissues. Briefly, tissue was homogenized in Trizol; nucleic acid was separated with chloroform at high speed centrifugation; and RNA was then precipitated with isopropyl alcohol and washed with 70% alcohol. Pellets of RNA were resuspended in DEPC water. The quality of RNA was assessed using an Agilent bioanalyzer 2100 (Agilent, Foster City, CA). RNA processing and microarray data generation was performed by the individual investigators at their own facilities. RNA processing protocols were generally those recommended by the Affymetrix microarray manufacturer.

For the current analysis, the subjects in the Neuropathology Consortium and the Array Collection were divided into two groups. Those with psychosis (N = 81) included those with schizophrenia and those with bipolar disorder with psychotic features. The non-psychotic controls (N = 82) included those with bipolar without psychotic features, those with depression, and the unaffected controls. Two cases with bipolar disorder were excluded due to insufficient information on psychotic features.

### Quality control of microarray data

All microarray raw data (cel files) were transformed using the Robust Multichip Average (RMA) normalization algorithm. The importance of quality control (QC) measures in microarray studies, including pre-chip (RNA quality of samples) and post-chip outcomes of the data, has been described previously [19]. We have performed a series of QC analyses to identify and remove microarray chip outliers before conducting statistical analysis on individual studies and on combined studies. Detailed QC procedures are described in our previous publications [20,21]. Briefly, each microarray chip was subjected to Affymetrix QC metrics for chip-level parameters such as scale factor, probe perfect match/mismatch difference counts, percent present calls, control gene (GAPDH and beta-actin) 5'/3' ratios and average correlation with respect to the reference distribution for those parameters across the arrays. Although no hard cutoffs were applied for each of the QC metrics, we examined the distribution of the metrics to determine whether samples appeared to be outliers.

### Analysis of microarray studies

The cross-study analysis includes 7 Affymetrix microarrays that were completed and provided to the SMRI from each investigator. The studies were coded as 1, 2, 3, 4, 5, 7 and 14 for the purpose of cross-study analysis to focus on the overall results and findings of the larger investigation, and also for the consistency with the SMRI online genomics database . Names of the investigators involved in microarray studies are as follows: Study 1: Altar A, Study 2: Altar C, Study 3: Bahn, Study 4: Chen, Study 5: Dobrin, Study 7: Kato, Study 14: Sklar A.

It is important to note that studies based on a common brain collection will have overlap in terms of subjects, thus the combined analysis on these studies are not completely independent. As a result, the degrees of freedom (df) can be over-estimated and the resulting p-values may be insufficiently stringent. In order to adjust for this lack of independence, a permutation method was used to identify a correction factor that adjusts the naïve df for the calculated p-value to a lower value (and subsequently higher p-value) at the point where the percentage of differentially expressed genes is equal to the selected alpha level (i.e. 5% differentially expressed genes found at p < 0.05) based on random data. This method used 7 microarray studies where both individual and cross-study analyses were computed for each iteration, for 100 total iterations, similar to our previous study [21].

For each iteration, the columns of each of 7 microarray data matrices were randomly shuffled to remove class memberships. Then, both individual study and cross-study analyses were computed on these permuted studies. Next, we calculated the cross-study p-values for the genes using df correction factor values starting at 0 and going up to 8 by increments of 0.2. For example, if the naïve df for gene X is 5, then using a correction value of 1.2, the adjusted df would be 3.8 and the adjusted p-value is calculated using this adjusted df. The number of genes at p < 0.05 was summated using each incremental df correction factor for 100 total permutations and the df (based on the naïve df minus the correction factor) that provided a median of 5% of the genes (p < 0.05) was selected. Genes where the difference between the naïve df and the correction factor was less than or equal to 0 were not counted in the gene percentages. This selected correction factor was then applied to the naïve df for each gene in the original data to provide adjusted p-values for each gene. We acknowledge the dependency between these studies where certain subjects are shared; however, after correcting for this over-estimated df, we found that our results are more powered to demonstrate consensus fold changes as compared to a single study.

### Individual study analysis

First, each demographic and clinical variable was assessed using a multiple regression model within each study. We identified probe sets that were significantly correlated with individual variables, where significance was defined as p < 0.01 and fold change >1.3 (the criteria that widely used in the previous brain microarray studies). For comparison of effect sizes, all demographics were analyzed using two levels. Continuous variables and ordered categorical variables were cut at values as close as possible to the median (e.g. continuous: PMI>30 vs. PMI<30; categorical: Heavy drug use vs. All lower levels of drug use) for the regression analysis. Demographic variables were assessed using both non-psychotic controls and psychosis subjects, while disease-specific variables were analyzed within the disease group to avoid the confounding of demographic effects and disease effects.

The following demographic variables were considered for all subjects: age, gender, PMI, brain pH, side of the brain, smoking at time of death, and sudden death. For subjects with psychosis, the following variables were considered: disease severity, heavy alcohol use, heavy drug use, suicide status, antipsychotic use, antidepressant use, and mood stabilizer use. Following the demographic analyses, the psychosis class was analyzed to identify a list of discriminating genes adjusted for the demographic terms (we used a common set of demographics for all analyses, including brain pH, PMI, lifetime antipsychotics, bipolar disorder, mood stabilizer lithium, lifetime alcohol, lifetime drug) or markers indicative of disease between the psychosis subjects and non-psychotic controls. The multiple regression analysis provided an adjusted fold change, standard error (SE), and p-value for each gene in each study.

### Cross-study analysis

The cross-study comparisons are based on scaled representations of individual study-level analysis across studies to extract the biological patterns and relationships. For the gene level analysis, consensus fold change was calculated for each gene based on a weighted combination of the individual fold changes and the SEs for the Affymetrix probe sets that map to each gene across the studies. Weights were determined in a probe set specific manner to account for the different levels of precision associated with each probe set that map to a given gene across the platforms. The weights were equal to 1/SE_i_, where SE_i _is the standard error of the ith probe set for the gene across all the studies. For the Gene Ontology (GO) analysis, GO association to disease was tested using a Fisher's Exact test (p < 0.05), based on the number of significantly regulated genes from the cross-study analysis within each GO term. Each GO term with number of genes between 10 and 500 were retained in the cross-study analysis. Additional information about cross-study analysis can be found at the SMRI online genomics database . The false discovery rate (FDR) was calculated based on the ratio of expected number of genes by chance at the specific p-value threshold (e.g. at p = 0.0001 and 19,502 genes = expect 2 genes by chance) divided by the sum of the expected number of genes by chance and the actual number of genes obtained at that threshold.

### Bioinformatics mappings

NCBI's Database for Annotation, Visualization and Integrated Discovery (DAVID 2007) was used as the standard source for gene annotation information [22]. The primary fields extracted from the DAVID include: Entrez ID, gene symbol, gene name and gene summary. Additional annotations include gene product mappings to the GO Consortium for the GO terms/classes. For microarrays, queries were based on the Affymetrix probe set ID (AFFYID).

### Real-time quantitative PCR

Total RNA was extracted from the dorsolateral prefrontal cortex tissues (BA46) of the Array Collection (psychosis N = 56, non-psychosis N = 49). RNA was further purified with the PureLink Micro to Midi Total RNA Purification System (Invitrogen, Carlsbad, CA) and the quality of RNA was assessed with the Bioanalyzer 2100 (Agilent, Foster City, CA). cDNA was synthesized with RT-PCR using oligo dT primers (Super Script III, Invitrogen, Carlsbad, CA). For real-time PCR, 1 μl aliquots of validated QuantiTect SYBR primer (Qiagen, Valencia, CA), 10 μl qPCR Master mix (Applied Biosystems, Foster City, CA), and 10 μl diluted cDNA were mixed together and pipetted into single wells of the qPCR plate (384-well format) with the Prism7900HT Sequence Detection System (Applied Biosystems, Foster City, CA). For no template controls for each gene tested, water was added instead of the cDNA. Thermo cycle conditions were: (1) 1 cycle for 2 min at 50°C, (2) 1 cycle for 15 min at 95°C, and (3) 40 cycles for 15 sec at 95°C and 1 min at 60°C and fluorescence was measured during the 60°C step for each cycle as recommended by the manufacturer. Reactions were quantified by the delta delta cycle threshold (Ct) method using SDS2.2 software (Applied Biosystems, Foster City, CA) generating a mean quantity value (Qty mean) for each sample from the triplicates of that sample for each gene. Recent studies have shown an advantage of using multiple endogenous control genes for the qPCR experiment [23], and therefore we used three endogenous control genes including β-2 microglobulin (B2M), glyceraldehyde-3-phosphate dehydrogenase (GAPDH) and β-actin (ACTB) in the qPCR experiment. The data for each gene of interest was expressed as Qty mean for the gene of interest/geometric mean of Qty mean values for the three endogenous control genes. Normalized values were then expressed as fold changes between the psychosis and the non-psychosis control group.

## Results

A summary of subject characteristics in 7 microarray studies is shown in Table 1. There were no significant differences in age, gender, race, brain pH and PMI between the psychosis and the non-psychosis group; this is important since these demographic and post-mortem variables appear to influence gene expression in the post-mortem brains. The psychosis subjects had a higher incidence of smoking at time of death, heavy drug use, heavy alcohol use, and suicide status compared to the non-psychosis controls, and these factors tend to be associated with psychotic features. Therefore, these psychosis-specific variables were included in the following demographic analysis.

**Table 1:** Summary of subject characteristics in the studies included in the cross-study analysis of psychosis

	Controls	Psychosis
# of Samples (Subjects)	213 (82)	220 (81)
Age	44.7+/- 9.8	43.9 +/- 10.2
Gender	70% Male	60% Male
Race	97% White	93% White
pH	6.49 +/- .32	6.34 +/- .28
PMI	31.0 +/- 15.8	32.9 +/- 15.7
Smoking at TOD	25%	57%
Heavy Drug Use	6%	23%
Heavy Alcohol Use	11%	31%
Suicide	11%	32%

For each variable, mean ± standard error or percentage value is reported. PMI: post-mortem interval, TOD: time of death

Table 2 represents the characteristics of individual studies, including the number of samples, brain collection, brain region, Affymetrix platform and number of probe sets in each study. Four studies (Study ID 1, 3, 5 and 7) used the Array Collection and three studies (Study ID 2, 4 and 14) used the Neuropathology Consortium. Note that total number of samples included in each study vary from 26 to 86. This is due to the availability of microarray raw data from each study and also due to the removal of poor quality arrays based on the QC analysis. Removal of microarray outliers was carried out before statistical analysis of individual and combined study analyses. Nonetheless, we validated a set of genes with the qPCR using a complete set of dorsolateral PFC tissues (N = 105) from the Array Collection.

**Table 2:** Summary of the seven microarray studies included in the cross-study analysis of psychosis

Study ID	Samples	Controls	Psychosis	Collection*	Region	Array Type	Probesets
1	81	38	43	A	Frontal BA46	Affy hgu133A	22283
2	55	30	25	C	Frontal BA46/10	Affy hgu133A	22283
3	86	42	44	A	Frontal BA46	Affy hgu133A	22283
4	26	16	10	C	Frontal BA6	Affy hgu133 2.0+	54681
5	73	35	38	A	Frontal BA46	Affy hgu133 2.0+	54681
7	81	35	46	A	Frontal BA46	Affy hgu133A	22283
14	31	17	14	C	Frontal BA8/9	Affy Hgu95Av2	12453

*A = Array Collection, C = Neuropathology Consortium

As an initial comparison across individual studies prior to the cross-study analysis, we used the significance criteria (p < 0.01 and fold change>1.3) that had been widely used in the previous microarray studies. The percentage of genes regulated by individual factors within each study is shown in Table 3. The median percentage of genes significantly regulated in psychosis is 0.62%, indicating the second highest percentage of genes significantly regulated. Brain pH was the most influential factor for gene expression with the median percentage of 1.28%. Other factors, including PMI (0.13%), heavy alcohol use (0.27%), antipsychotic use (0.28%) and lithium use (0.22%), also showed comparable number of gene expression changes in this analysis.

**Table 3:** Demographic and clinical variable analysis in individual studies

Factor	Median % Regulated in Individual Studies
Psychosis	.62%
Smoking	.03%
Gender	.12%
PMI	.13%
Brain Side	.03%
Brain pH	1.28%
Age	.03%
Heavy Alcohol Use	.27%
Heavy Drug Use	.12%
Suicide	.05%
Agonal State (Sudden Death)	.19%
Antidepressant Use	.04%
Antipsychotic Use	.28%
Mood Stabilizer Use	.09%
Lithium Use	.22%
Valproate Use	.07%

Median percentage of genes regulated in individual studies is shown with each variable. The criteria of significance (p-value < 0.01 and FC >1.3).

Following the analyses on individual studies, we performed cross-study analysis to identify a list of discriminating genes in psychosis. Table 4 represents the summary results of the cross-study analysis including fold changes and p-values for all genes. Most genes showed small fold changes (less than 1.3) in this analysis. The cumulative FDR analysis indicates that a p-value cutoff of 0.001 would maximize the number of genes while keeping the FDR relatively low (14%). For example, 110 genes showed a highly significant change at p < 0.001 (see Additional file 1). The number of genes found to be differentially expressed while maintaining a relatively low FDR illustrates the robust expression differences between individuals with and without psychosis.

**Table 4:** Summary of cross-study analysis of psychosis showing fold changes and p-values.

Psychosis Combined Analysis							
			p-value				
Fold Change	<.0001	.0001–.0005	.0005–.001	.001–.005	.005–.01	.01–.05	>.05
1–1.1	28	40	26	184	197	1327	17343
1.1–1.2	5	4	3	18	14	73	192
1.2–1.3	1	0	2	2	1	5	31
>1.3	0	1	0	0	0	0	5
Total	34	45	31	204	212	1405	17570
Chance	2	7	9	72	90	725	18527
							
Cum total	34	79	110	314	526	1931	19502
Cum chance	2	9	18	90	180	906	19502
Cum FDR	5.6%	10.2%	14.1%	21.8%	25.5%	31.9%	---

Cum total: Cumulative total, Cum FDR: Cumulative false discovery rate

**Figure 1:**
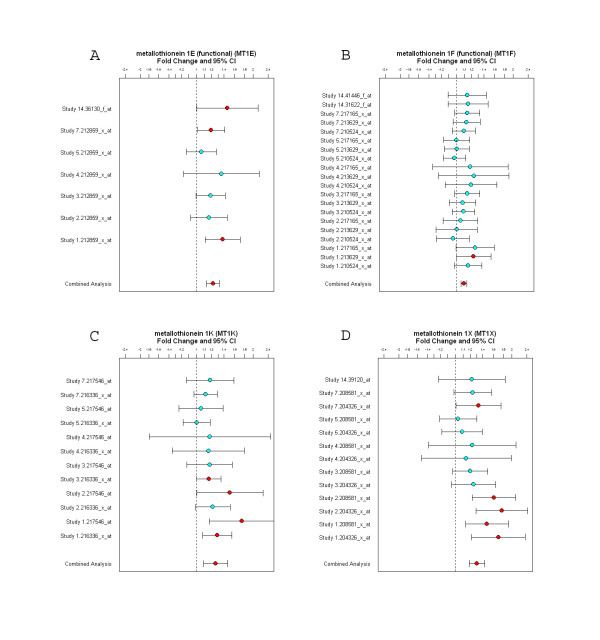
**Increased gene expression of metallothioneins in the cross-study analysis of psychosis**. A: metallothionein 1E, B: metallothionein 1F, C: metallothionein 1K, D: metallothionein 1X. The plots with fold changes and 95% confidence intervals show consistent up-regulation of metallothioneins across individual studies as shown on the Y-axis. Combined analysis shown on the bottom of each panel represents the weighted fold change and 95% confidence intervals for each gene.

From the cross-study analysis, we have identified the genes that are differentially regulated in psychosis compared to non-psychosis using a relatively large fold change and moderate p-value threshold (p < 0.05). Such guidelines have been suggested by the MicroArray Quality Control (MAQC) I project to maximize reproducibility of differential expression across platforms [24]. Additionally, large fold change magnitudes are preferred for validation purposes on an alternative platform such as qPCR. Among differentially regulated genes, a set of metallothionein genes are consistently up-regulated across the studies (Figure 1). The p-values and fold changes for the genes are MT1E (p = 0.002, FC 1.22), MT1F (p = 0.00005, FC 1.1), MT1K (p = 0.027, FC 1.26) and MT1X (p = 0.0001, FC 1.31). As shown at the bottom of each graph, the combined analysis with weighted fold change and 95% confidence intervals indicates significant expression changes. For instance, MT1F gene is not significant in most individual studies, however, the combined analysis clearly shows that MT1F gene is significantly up-regulated because of the increased power to detect small but consistent changes across multiple studies (Figure 1B).

**Figure 2:**
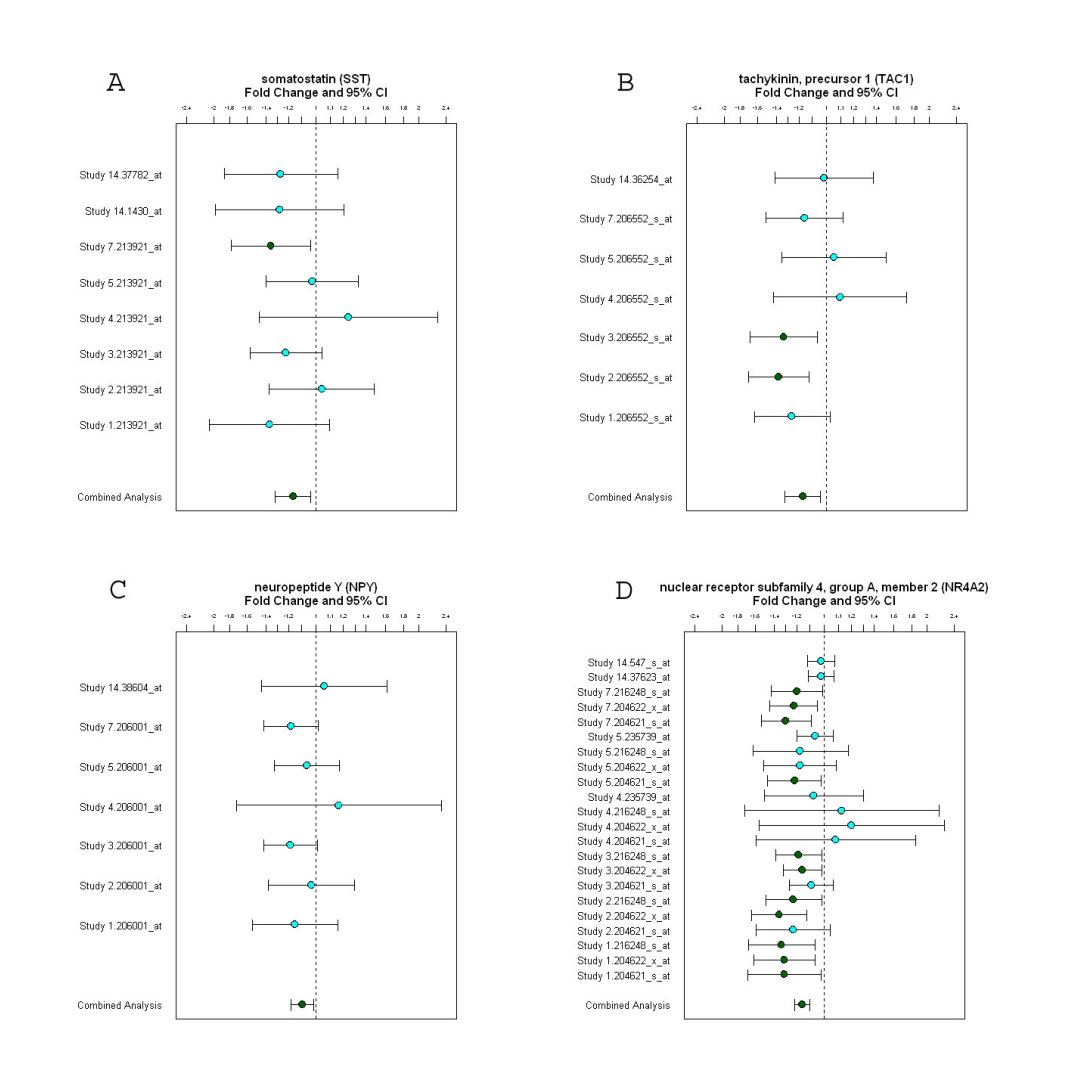
**Decreased gene expression of neuropeptides in the cross-study analysis of psychosis.** A: somatostatin, B: tachykinin, precursor 1, C: neuropeptide Y, D: NR4A2. The plots with fold change and 95% confidence intervals show consistent down-regulation across multiple individual studies. Combined analysis shown on the bottom of each panel represents the weighted fold change and 95% confidence intervals for each gene.

Down-regulated genes in psychosis include several neuropeptide genes (Figure 2). The p-values and fold changes for the genes are somatostatin (SST) (p = 0.038, FC -1.17), Tachykinin precursor 1 (TAC1) (p = 0.04, FC -1.17), and neuropeptide Y (NPY) (p = 0.05, FC -1.09). A nuclear receptor subfamily 4, group A, member 2 (NR4A2) gene was also down-regulated (p = 0.00001, FC -1.16) in psychosis. This gene has been implicated in psychiatric disorders including schizophrenia and bipolar disorder. Most individual studies show consistent down-regulation of NR4A2 gene, except the study 4, and the combined analysis at the bottom indicates that NR4A2 gene is significantly down-regulated in psychosis (Figure 2D).

**Figure 3:**
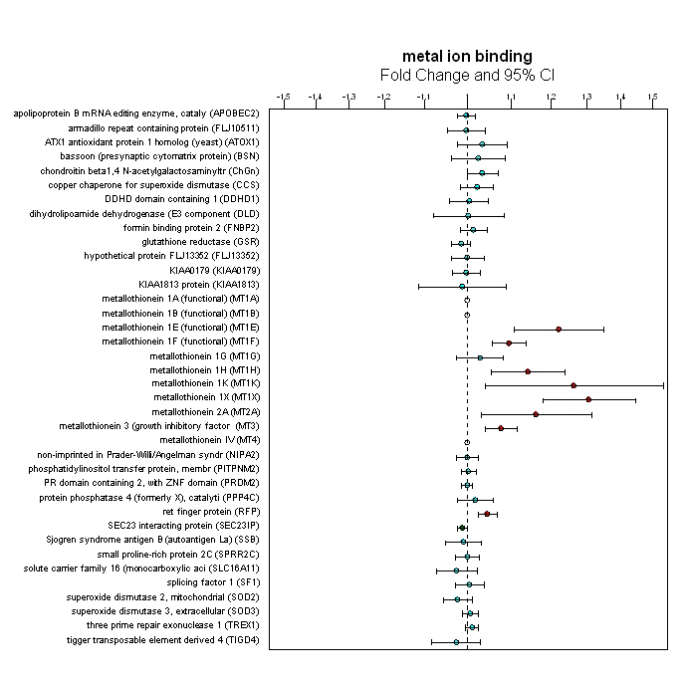
**Consistent up-regulation of metallothionein genes among metal ion binding genes in the Gene Ontology (GO) term**. Seven metallothionein genes including MT1E, MT1F, MT1H, MT1K, MT1X, MT2A and MT3 are significantly up-regulated in this category.

The GO analysis of metal ion binding function is shown in Figure 3. The metallothionein genes including MT1E (p = 0.002, FC 1.22), MT1F (p < 0.0001, FC 1.1), MT1H (p = 0.007, FC 1.14), MT1K (p = 0.027, FC 1.26), MT1X (p = 0.0001, FC 1.31), MT2A (p = 0.023, FC 1.16), and MT3 (p = 0.001, FC 1.08) are constantly up-regulated as compared to other genes associated with the metal ion binding function. Metal ion binding was found to be among the top 9 GO terms significantly regulated in the cross-study analysis of psychosis (p = 0.01) and was chosen to highlight based on the robust and consistent changes in a shared gene group (i.e. metallothionein genes). Additional significant GO terms include: regulation of cell growth (p = 0.001), negative regulation of transcription from Pol II promoter (p = 0.003), voltage-gated potassium channel activity (p = 0.005), metabolism (p = 0.006), translational elongation (p = 0.006), learning and/or memory (p = 0.006), WNT receptor signaling pathway (p = 0.008), and glutathione transferase activity (p = 0.009) (see Additional file 2).

**Figure 4:**
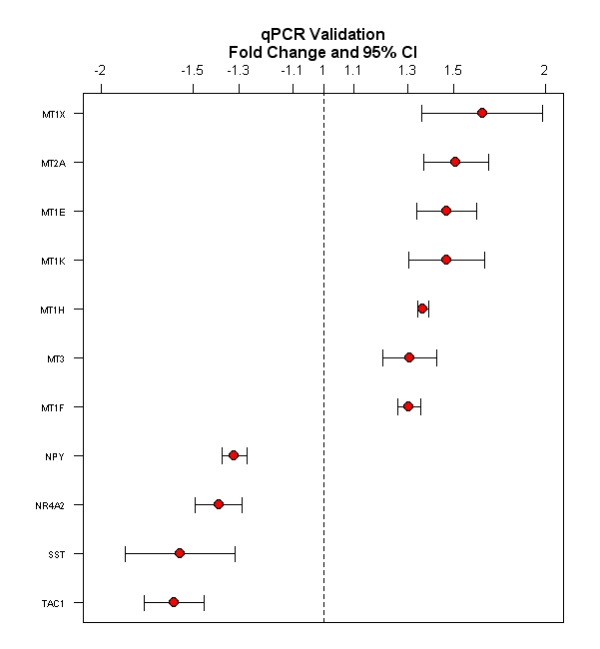
**Quantitative PCR validation of metallothionein and neuropeptide genes**. The gene plots with fold changes and 95% confidence intervals show that metallothionein genes including MT1X, MT2A, MT1E, MT1K, MT1H, MT3, AND MT1F are significantly up-regulated and four genes including NPY, NR4A2, SST and TAC1 are significantly down-regulated in psychosis (p < 0.05).

Following the cross-study analysis of microarrays, we performed a qPCR experiment to validate gene expression changes that we observed in microarrays (Figure 4). We confirmed differential expression of metallothionein genes including MT1X (p = 0.001, FC 1.64), MT2A (p = 0.009, FC 1.51), MT1E (p = 0.01, FC 1.47), MT1K (p = 0.005, FC 1.47), MT1H (p = 0.03, FC 1.36), MT3 (p = 0.004, FC 1.3) and MT1F (p = 0.02, FC 1.3). The order of fold changes of metallothionein genes in the qPCR corresponds to the order of fold changes of these genes observed in the microarray analysis. We have also confirmed that expression of the neuropeptide genes are down-regulated in psychosis; NPY (p = 0.02, FC -1.32), NR4A2 (p = 0.01, FC -1.39), SST (p = 0.001, FC -1.57) and TAC1 (p = 0.01, FC -1.6). Overall fold changes of these genes that we found with the qPCR experiment are larger than fold changes observed in the microarray analysis.

## Discussion

In the current study, we have found the genes that are differentially regulated in the prefrontal cortex of individuals with psychosis. These findings are important because we performed a cross-study analysis of seven microarray studies that used three different Affymetrix platforms with two different post-mortem brain collections. Therefore, these gene expression changes are "consensus" fold changes across multiple microarray studies following adjustment of confounding variables in individual studies.

It is well known that gene expression studies of psychiatric disorders using post-mortem brains are more challenging than others [25-28]. Possible reasons for this complexity include pre- and post-mortem factors such as RNA quality, brain pH, PMI and agonal state that affect gene expression patterns in the brains of psychiatric patients [23]. Moreover, biological and disease-specific effects are often hindered by several issues such as relatively small sample sizes, small effect sizes and heterogeneity of psychiatric phenotypes in the general population. The clinical information available for the patients is typically sparse, so that unknown clinical covariates may either confound or confuse many of the gene expression findings in psychiatric disorders [20]. Therefore, appropriate statistical adjustment using such demographic and clinical information is critical in order to improve inferences in determining putative genes and biological pathways in psychiatric disorders.

Gene expression microarray technology has become a powerful tool for discovery, although this technology had some limitations such as high cost of experiments, limited sample size, and lack of standardization on data analysis. Recently, there have been improvements in the microarray field, and more microarray datasets are available for meta-analysis. Several studies have described important issues related to the meta-analysis of microarray data including standardization, statistical analysis and databasing strategy [29], and the development of new bioinformatic tools such as searchable online archives of genetic/genomic findings in psychiatric disorders [30]. One of the main advantages of meta-analysis as compared to an individual study is the increased statistical power to detect consensus fold changes across multiple studies. It has been reported that gene expression changes in post-mortem brains of psychiatric patients are relatively small. This may explain some of the discrepancies in previous studies that reported putative genes in psychiatric disorders, because most of the studies were not sufficiently powered (due to small sample sizes) to detect small changes in gene expression. Moreover, some studies did not have sufficient clinical information of post-mortem brains to adjust for possible confounding variables.

In the current analysis, we have included seven microarray studies that used the frontal cortex (BA46/10, BA8/9 and BA6) as compared to the previous study [21] that included the cerebellum studies. A recent study demonstrated that different brain regions such as the frontal, cingulate, temporal, parietal, occipital cortices, hippocampus and sub-cortical regions show different patterns of gene expression [31]. The authors also found that gene expression patterns in the prefrontal cortices (BA10 and BA46) are similar to each other as compared to non-frontal regions. To our knowledge, there is little evidence on gene expression differences in the sub-regions of the frontal cortex because most studies have focused on one region of the frontal cortex of schizophrenia patients. Another limitation was an availability of the prefrontal cortex (BA46) tissues from the Neuropathology Consortium when individual microarray studies were conducted. This brain cohort has been used by many investigators for various types of research in the past. Therefore, we included 3 studies that used the frontal cortices (BA46/10, BA8/9 and BA6) from the Neuropathology Consortium because these regions are adjacent to each other and have been implicated in schizophrenia [32-34]. Nevertheless, we included another 4 studies that used the Array Collection with the same dorsolateral prefrontal cortex (BA46).

In the current cross-study analysis, we identified a set of metallothionein genes including MT1X, MT1K, MT1E, MT1H, MT1F, MT2A and MT3 that are significantly up-regulated in psychosis. We used a real-time qPCR to validate differential expression of metallothionein genes in the prefrontal cortex (BA46) of psychosis patients. Real-time qPCR is often referred as the gold standard for validation of gene expression in microarrays due to its advantages in detection sensitivity, large scale dynamic range, high precision and reproducible quantitation compared to other techniques [35,36]. For the qPCR, we used a complete set of dorsolateral prefrontal cortex (BA46) tissues from the Array Collection (psychosis N = 56, non-psychosis N = 49). It is important to note that most previous microarray studies have used a relatively small number of post-mortem brains for the qPCR validation.

Although metallothioneins were discovered almost 50 years ago, their functional role in the brain has not been well-characterized. Putative physiological functions of metallothioneins in the CNS, including neuroprotection, regeneration and cognitive function, have been described in a recent paper [37]. Other studies reported that metallothioneins are involved in cellular host defense response, stress response, immunoregulation, cell survival and brain repair [38-41]. Astrocytes appear to be the main source of metallothioneins in the brain, even though the primary function of metallothioneins are protecting neurons from a variety of pathology [42]. Increased MT1 and MT2 gene expression have been reported in several types of brain pathology including traumatic and excitotoxic injury, amyotrophic lateral sclerosis, Alzheimer's disease and Parkinson's disease [43-47]. Studies using animal models showed that metallothioneins may be involved in behavior associated with substance dependence [48] and learning and memory [49]. Similar to our findings, one study reported that MT2A gene expression is increased in the PFC of schizophrenia patients [50]. These results suggest that metallothioneins in the brain may play a significant role in neuroprotection and cognitive function. It is possible that neurodegenerative process may disrupt cognitive function in the frontal cortex of psychosis patients and up-regulation of metallothionein genes in the PFC may be a compensatory mechanism against these adverse processes. Interestingly, a recent study reported that metallothionein-related compounds are well tolerated in animal studies [39], and therefore, these compounds may be potential candidates for novel medication development in psychosis-related disorders.

The genes down-regulated in psychosis include neuropeptide genes such as somatostatin (SST), neuropeptide Y (NPY) and tachykinin (TAC1). The neuropeptide genes have been implicated in working memory function and in schizophrenia [51]. The NPY gene may be associated with schizophrenia [52], impulsivity [53], aggression and bipolar disorders [54,55]. Previous studies reported decreased NPY levels in the temporal cortex of schizophrenia [56] and decreased NPY receptor gene expression in lymphocytes of schizophrenia [57]. Gabriel et al reported that both NPY and SST levels are decreased in the cerebral cortex of individuals with schizophrenia [58]. A microarray study reported that SST gene expression is increased in the PFC of bipolar disorder but not with schizophrenia patients [59]. It is possible that this study may not have been sufficiently powered (due to small sample sizes) to detect small gene expression changes in schizophrenia. Consistent to the current results, mRNA levels of SST in the PFC are decreased in schizophrenia patients in a study using *in situ *hybridization [60] and another microarray study reported that both SST and NPY expression levels are decreased in the PFC of schizophrenia [51]. TAC1-related genes are also implicated in psychiatric disorders [61,62] and increased protein levels of TAC1 receptor in the PFC of schizophrenia have been reported [63]. Taken together, previous findings and the current results provide strong evidence for involvement of neuropeptide genes in psychosis-related disorders.

One of the potential confounding variables affecting neuropeptide gene expression may be the antipsychotic medication. Previous studies reported that NPY levels in the brain are affected by various drugs including typical and atypical antipsychotics [64,65]. Using rodents, one study reported that NPY and SST levels are increased in the PFC following haloperidol treatment [66], while another study reported that NPY levels are decreased following chronic antipsychotic treatment [67]. However, GABA-system related genes including SST are not changed in the PFC of monkey brains treated with chronic antipsychotic drugs [51]. In order to minimize the confounding effect of antipsychotic medication from the disease effect, we used multiple regression models to adjust antipsychotic medication in the current analysis.

Another gene down-regulated in psychosis is the transcription factor Nurr1 (NR4A2), an orphan nuclear receptor associated with the development of dopaminergic cells in the mid-brain (p < 0.00001, FC-1.16). This gene has been implicated in attention deficit hyperactivity disorder, alcohol dependence, schizophrenia and bipolar disorder [68-70]. A recent study with NR4A2 knock-out mice suggests that these animals display behavioral endophenotypes that are displayed in other animal models of psychosis/schizophrenia. [71]. Therefore, reduced NR4A2 gene expression in the PFC may be associated with psychotic features.

Although genome-wide gene expression profiling in the postmortem brains may reveal valuable information related to psychiatric disorders, this approach alone is limited in terms of being able to distinguish between changes reflecting the primary disease etiology from those reflecting compensatory mechanisms and many potential confounding influences such as medications and drug use [72]. Therefore, it is necessary to study the relationship between gene expression, genetic and epigenetic variations in individuals with psychiatric disorders. The integration of postmortem gene expression with genetic variations including single nucleotide polymorphisms (SNPs) is the subject of ongoing investigations.

## Conclusion

We found a set of genes that are differentially regulated in the prefrontal cortex of individuals with psychosis. Among those genes, metallothionein genes are consistently up-regulated and neuropeptide genes are down-regulated. These genes may have implications in neurodegeneration and working memory function in the prefrontal cortex that are thought to be disrupted in psychosis. With an advantage of cross-study analysis, we were able to detect small but "consensus" changes in gene expression across multiple microarray studies. By combining gene expression analyses across multiple microarray studies and with the qPCR validation of putative genes, we can provide greater confidence in the scientific findings, which include potential genes and biological pathways in psychiatric disorders, as compared to the result obtained from a single study.

## Competing interests

The authors declare that they have no competing interests.

## Authors' contributions

KC conception and design, analysis and interpretation of data, drafting the manuscript. ME and BH conception and design, analysis and interpretation of data, revisions of the manuscript. JS, SK, SS and SD acquisition of data, analysis and interpretation of data. RY, MK, EFT and MW analysis and interpretation of data, revisions of the manuscript.

## Pre-publication history

The pre-publication history for this paper can be accessed here:



## Supplementary Material

Additional file 1 **A list of transcripts that shows significant regulation in the cross-study analysis of psychosis (p < 0.001)** Click here for file

Additional file 2 A list of GO terms that shows enriched gene sets in the cross-study analysis of psychosis (p < 0.05) Click here for file
